# Unerwünschte Ereignisse bei Patienten unter Immuntherapie nach Strahlentherapie – gepoolte Analyse von Studien aus der Datenbank der US Food and Drug Administration

**DOI:** 10.1007/s00066-022-01919-0

**Published:** 2022-03-31

**Authors:** Paul Griebel, Jürgen Dunst

**Affiliations:** Klinik für Strahlentherapie, Universitätsklinikum Schleswig-Holstein, Campus Kiel, Feldstr. 21, 24105 Kiel, Deutschland

## Hintergrund und Ziele

Der klinische Erfolg von Immuncheckpointinhibitoren (ICI) hat die Immuntherapie – neben der Chirurgie, Chemo- und Radiotherapie (RT) – als vierte Säule der Krebsbehandlung etabliert. Im Vergleich zu konventionellen Chemotherapeutika weisen ICI ein eigenes Spektrum an Nebenwirkungen auf, das sich vor allem durch Überaktivierung und Toleranzverlust des Immunsystems erklären lässt [1]. Diese Therapeutika werden aktuell noch hauptsächlich in palliativer Indikation und – aufgrund hoher Kosten – meist als Zweit- bzw. Drittlinientherapie eingesetzt. Ein Großteil der Patienten hat zu diesem Zeitpunkt bereits eine Strahlentherapie erhalten. Durch die Freisetzung von Stresssignalen und Neoantigenen wird der Bestrahlung ebenfalls eine immunmodulierende Funktion zugesprochen [2, 3]. Solche Effekte haben eine lange Halbwertszeit. Biologisch ist es also wahrscheinlich, dass eine Abfolge der beiden Therapien verstärkt immunogen wirkt. In Zukunft muss das Risiko-Nutzen-Verhältnis einer therapeutisch erhofften Synergie mit einer Verstärkung von adversen Effekten bedacht werden [4].

Im klinischen Alltag werden allerdings bereits heute zahlreiche Patienten mit kurz zuvor erfolgter Radiotherapie auf eine Immuntherapie eingestellt. Da eine enge zeitliche Abfolge jedoch ein Ausschlusskriterium der meisten ICI-Zulassungsstudien war, fehlen derzeit noch für die Behandlungsplanung Daten, um die Sicherheit dieser Sequenz zu optimieren. Anscher et al. [5] analysierten daher die Metadaten aus Zulassungsstudien, um eine erste Einschätzung bezüglich veränderter Nebenwirkungsprofile treffen zu können.

## Patienten und Methoden

Die Autoren kombinierten Daten aus 68 klinischen Studien zwischen Februar 2003 und Dezember 2019. Sie trennten eine Gruppe von Patienten mit einer RT vor mehr als 90 Tagen (> 90 d) von einer Kohorte mit einer RT innerhalb von 90 Tagen (≤ 90 d) vor Beginn des ICI, da sich in dieser Zeitspanne nach Therapieabschluss noch akute Strahlenreaktionen manifestieren können. Mithilfe eines „propensity score“ [6] stellten sie beiden Gruppen eine gleiche Anzahl an ICI-Patienten ohne vorherige RT gegenüber. Verglichen wurden die Gruppen im Hinblick auf typische ICI-assoziierte Nebenwirkungen.

## Ergebnisse

Von 25.469 infrage kommenden Patienten wurden 16.875 in die Analyse eingeschlossen. Kriterien waren die erfolgte Behandlung mit einem Checkpointinhibitor und das Vorhandensein aller Variablen, welche in der Propensity-Analyse berücksichtigt worden waren. Davon erhielten 13.956 (83 %) keine vorherige RT, 1146 (6 %) > 90 d und 1773 (11 %) ≤ 90 d. Der Großteil der Patienten war jünger als 65 (56,1 %), männlich (62,1 %), weiß (79,7 %) und wurde in den USA behandelt (40 %). Die häufigsten Primärtumoren gingen von Lunge, Haut, Niere, Oropharynx und Blase aus.

Es zeigten sich in allen Vergleichen keine Unterschiede in den Nebenwirkungen von Grad 3–4 für Patienten mit oder ohne vorherige Radiotherapie. Vor jeglicher Anpassung der Stichprobenverteilung zeigten Patienten mit RT ≤ 90 d häufiger Nebenwirkungen vom Grad 1–2, und zwar in Form von Fatigue, Endokrinopathien und Pneumonitis. Bei einer RT > 90 d wurde einzig eine höhere Rate an Low-grade-Pneumonitiden festgestellt.

Nach Normalisierung der Vergleichsgruppen mittels des „propensity score“ zeigte sich weiterhin eine höhere Inzidenz von Pneumonitiden bei allen Patienten mit einer vorangegangenen RT. Erfolgte diese nach einem Intervall ≤ 90 d, zeigte sich zusätzlich eine Häufung von Fatigue, Thrombozytopenie und renalen Nebenwirkungen, während unerwünschte dermatologische Effekte seltener waren. Weiterhin machte eine RT nach ≤ 90 d einen Therapieabbruch aufgrund von Pneumonitis häufiger als ohne RT notwendig. Ein Therapieabbruch in der > 90 d-Gruppe war interessanterweise seltener.

Im Vergleich zwischen den gängigsten Checkpointinhibitoren untereinander zeigte eine vorherige RT gegenüber der Kontrollgruppe verschiedene Effekte. Während eine Kombinationstherapie mit Anti-PD‑1 und Anti-CTLA‑4 häufiger Kolitiden machte, waren Endokrinopathien seltener. Unter einer PD-L1-Therapie waren dagegen Endokrinopathien und Pneumonitiden häufiger. Bei allen Präparaten war Fatigue nach RT gehäuft.

## Schlussfolgerungen der Autoren

Die Autoren stellen fest, dass in keiner der 68 analysierten Studien ein Zusammenhang besteht zwischen der Häufigkeit schwerer Nebenwirkungen unter Immuntherapie nach vorangegangener Radiotherapie. Sie erkennen jedoch eine Häufung von geringgradigen Nebenwirkungen wie Fatigue, Endokrinopathie und Pneumonitis, wenn die RT ≤ 90 Tage zurücklag.

## Kommentar

Viele Patienten werden in engem zeitlichem Zusammenhang mit dem Beginn einer Immuntherapie bestrahlt, häufig in palliativer Indikation, z. B. bei schmerzhaften Knochenmetastasen. Diese Kombination wird aus Notwendigkeit und aufgrund fehlender aussagekräftiger Daten mehr oder weniger unkritisch durchgeführt. Prospektive Daten bezüglich der Sicherheit einer solchen Behandlungsabfolge sind rar.

Anscher und Kollegen ziehen nun in ihrer Studie aus bereits vorhandenen Datensätzen erste klinisch relevante Schlüsse und überbrücken damit das vorherrschende Wissensvakuum. Die Arbeit hat jedoch einige Limitationen, die für behandlungsrelevante Schlussfolgerungen bedacht werden sollten. Erstens umfasste die Studie Patienten mit verschiedenen Grunderkrankungen, die unterschiedlich behandelt wurden. Nebenwirkungen sind meist organspezifisch und damit entscheidend von der Lokalisation der Bestrahlung abhängig. Es gibt zusätzlich Grund zu der Annahme, dass Dosis und Fraktionierung der RT Einfluss auf den immunmodulatorischen Effekt der Bestrahlung haben [2].

Zweitens wurden in den Zulassungsstudien keine Details bezüglich der Radiotherapie erfasst, was die nicht zufriedenstellende binäre Einteilung der Patienten in „RT“ und „keine RT“ erforderlich machte. Die dadurch verursachte Datenunsicherheit sorgt für differente Ergebnisse je nach Betrachtungsmethode. Dennoch lassen sich aus der Analyse dieser Metadaten vorsichtige Schlüsse für die klinische Praxis ziehen:

- Der Großteil der Patienten war von Lungenkrebs befallen, mit über 30 % in der ≤ 90 d- und über 50 % in der > 90 d-Gruppe. Man kann annehmen, dass die RT in dieser Population häufig das Lungengewebe der betreffenden Patienten miterfasste. Radiogene Reaktionen an der Lunge treten oft erst Monate nach Therapie auf [7]. Es ist plausibel anzunehmen, dass eine Immuntherapie diese entzündlichen Prozesse verstärkt hat. Dies erklärt die Häufung von Pneumonitiden in beiden Post-RT-Gruppen, und diese Nebenwirkung rechtfertigt die Entscheidung, die Therapie abzubrechen. Vor allem kurzfristig nach einer Strahlenbehandlung der Lunge sollte das Nutzen-Risiko-Verhältnis einer Therapie mit Checkpointinhibitoren sorgfältig abgewogen werden. Im Umkehrschluss muss man bei diesem Patientenkollektiv in Antizipation einer möglichen späteren ICI-Therapie diesen Aspekt bereits bei der Bestrahlungsplanung berücksichtigen.
- Eine Erklärung für die vereinzelt beobachtete höhere Rate von Thrombozytopenien bei fehlender Häufung von Neutropenien in der ≤ 90 d-Gruppe bleibt offen. Theoretisch könnte eine Bestrahlung des Knochenmarks über eine Immunsuppression die Wirksamkeit eines ICI negativ beeinflussen. Eine klinische Relevanz ist unwahrscheinlich.
- Dass sich schwere Nebenwirkungen der ICI-Therapie nach Bestrahlung nicht häufen, spricht für eine ausreichende Sicherheit in der aktuellen, heterogenen klinischen Praxis. Es ist jedoch zu vermuten, dass die Immunogenität der Bestrahlungsmodalitäten direkt mit der Wahrscheinlichkeit von höhergradigen Nebenwirkungen korreliert ist. Aktuelles Wissen über den Effekt von Dosis, Lokalisation und Fraktionierung auf das Immunsystem sollte also in die Behandlungsplanung mit eingehen.

## Fazit

Eine Therapie mit Immuncheckpointinhibitoren in kurzer Abfolge nach einer Radiotherapie ist oft klinisch notwendig. In der hier vorgestellten Analyse von über 2900 Strahlentherapiepatienten aus 68 verschiedenen Studien mit Immuntherapie ergab sich bisher kein Hinweis für ein erhöhtes Risiko höhergradiger Nebenwirkungen dieser Kombinationstherapie. Geringgradige Toxizitäten können häufiger auftreten, zwingen aber selten zum Therapieabbruch. In prospektiven Studien sollten künftig Details der Radiotherapie, wie Dosis, Fraktionierung und Lokalisation, besser miterfasst und mitgeteilt werden.

*Cand. med. Paul Griebel, Kiel und Prof. Dr. Jürgen Dunst, Kiel*
